# Ethical Integration of Artificial Intelligence in Healthcare: Narrative Review of Global Challenges and Strategic Solutions

**DOI:** 10.7759/cureus.84804

**Published:** 2025-05-25

**Authors:** Madhusudan P Singh, Yogendra N Keche

**Affiliations:** 1 Pharmacology, All India Institute of Medical Sciences, Raipur, Raipur, IND

**Keywords:** accountability, artificial intelligence in healthcare, bioethics, data privacy, ethical frameworks, machine learning, patient safety

## Abstract

Artificial intelligence (AI) is revolutionizing healthcare, offering innovative solutions to enhance diagnostic accuracy, treatment efficacy, and accessibility. However, integrating AI into clinical practice raises significant ethical implications, necessitating clear guidelines and frameworks. This paper provides a comprehensive review of the ethical landscape surrounding AI deployment in healthcare across various countries and regions. It explores AI's transformative potential while underscoring the need to prioritize patient safety, transparency, accountability, data privacy, fairness, and human oversight. The paper analyzes existing guidelines from the European Union, the United States, India, Australia, and Africa, identifying common ethical principles and considerations. It discusses challenges and proposes suggestions for addressing issues such as data quality and bias, transparency, privacy and security, accessibility and equity, and robust legal and regulatory frameworks. By fostering a comprehensive understanding of the ethical implications and guidelines surrounding AI in healthcare, this paper aims to contribute to the responsible development and equitable deployment of these transformative technologies.

## Introduction and background

Artificial Intelligence (AI) is rapidly transforming healthcare by enabling more precise diagnostics, predictive analytics, and personalized treatment plans. Among the core subtypes of AI, machine learning (ML) involves training algorithms to detect patterns in large datasets, widely used for predictive modeling in disease risk stratification. Deep learning, a subset of ML, employs neural networks to process complex data such as medical imaging, achieving performance comparable to human experts in fields like radiology and pathology. Natural Language Processing (NLP) allows AI systems to interpret and generate human language, enabling applications such as clinical documentation, chatbot-based symptom checkers, and the extraction of insights from electronic health records [1].

However, the ethical integration of AI into healthcare presents unique challenges compared to other sectors. Unlike consumer-facing AI, medical AI directly influences patient safety, clinical decision-making, and health outcomes. The use of sensitive personal health data, potential algorithmic biases, lack of transparency in decision-making (“black box” models), and uneven access to AI tools raise significant concerns about privacy, fairness, accountability, and trust. These factors necessitate a robust ethical framework to guide the responsible development, implementation, and oversight of AI technologies in healthcare systems globally [1].

The ethical integration of AI into healthcare is becoming increasingly critical, not only because AI technologies are growing more sophisticated and widespread, but also due to the high stakes involved in patient safety, data privacy, regulatory compliance, and the need to build and maintain public trust. This review explores the evolving bioethical landscape of AI in healthcare, emphasizing its transformative potential and the urgent need for structured ethical frameworks to guide its development and application. To provide context, this review introduces key AI subtypes, ML, deep learning, and NLP, and illustrates their application in clinical settings. ML is widely used for predictive analytics, while deep learning enables advanced image recognition in diagnostics. NLP facilitates the interpretation of unstructured clinical data and supports automated documentation and decision-making tools. Healthcare poses unique ethical challenges for AI compared to other fields, including the use of sensitive health data, potential algorithmic bias, opaque decision-making processes, and unequal access to technology. These complexities necessitate deliberate and inclusive governance [1-3].

In response, this review incorporates the most recent global policy documents, including the 2024 updates to the European Union’s AI Act, which outlines a risk-based regulatory framework for high-stakes applications, and the WHO’s 2021 guidance on AI ethics, which promotes transparency, inclusiveness, and human oversight. By analyzing ethical guidelines from diverse regions, including the European Union, the United States, India, Australia, and Africa, this paper identifies common principles such as accountability, fairness, and privacy, and critically examines the challenges associated with their implementation in real-world healthcare systems [1-3].

As AI reshapes clinical practice, ethical concerns surrounding patient safety, transparency, accountability, data privacy, fairness, and human oversight have become increasingly prominent. Addressing these concerns requires not only robust legal frameworks but also a deep understanding of bioethical principles. This review aims to facilitate this understanding and promote a responsible, inclusive, and sustainable integration of AI in global healthcare systems.

Methodology

This review adopts a narrative methodology informed by a systematic approach to literature sourcing, with the goal of synthesizing and critically interpreting ethical frameworks surrounding the use of artificial intelligence (AI) in healthcare. While this is not a formal systematic review, it incorporates systematic elements in the identification, selection, and thematic analysis of relevant literature to enhance rigor and transparency.

A non-structured but purposive search strategy was employed across multiple databases, including PubMed, Scopus, and Google Scholar. The search involved various combinations of keywords such as “AI in healthcare,” “ethical frameworks,” “bioethics and artificial intelligence,” and “AI governance.” The aim was to identify a wide range of guidelines, frameworks, and scholarly literature relevant to the ethical and governance aspects of AI in healthcare.

Documents were selected based on their relevance to the healthcare context, their focus on ethical or governance issues, and their publication by authoritative bodies such as the World Health Organization (WHO), the European Union (EU), the U.S. Food and Drug Administration (FDA), and the Indian Council of Medical Research (ICMR). Only documents published between 2018 and 2024 were included to ensure contemporary relevance. Literature that did not pertain directly to healthcare applications of AI, such as general or non-healthcare AI ethics guidelines, as well as opinion articles lacking policy relevance and duplicate content, were excluded from the analysis.

**Figure 1:**
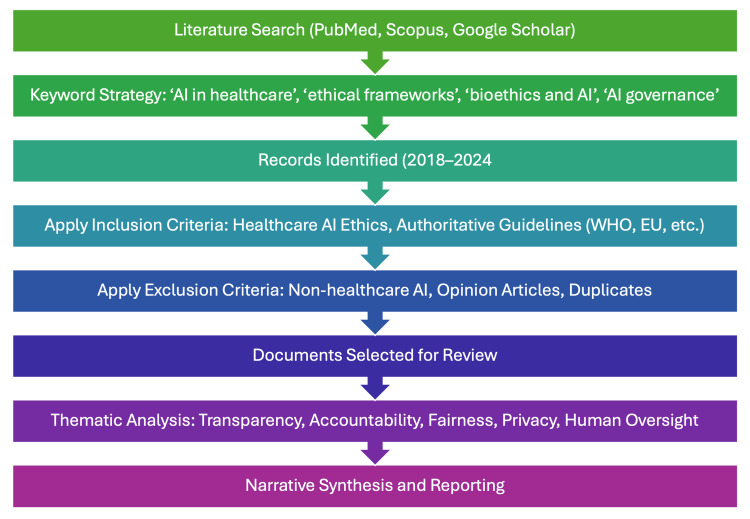
Study flowchart Created by the authors.

The included literature and guidelines were then analyzed thematically. Thematic synthesis focused on identifying common and divergent elements across key ethical dimensions, including transparency, accountability, data privacy, fairness, and human oversight. This hybrid approach allowed for structured identification and selection of literature while maintaining the flexibility of a narrative review to interpret and contextualize the findings within a broader ethical and healthcare landscape (Figure 1).

## Review

What is AI in healthcare?

AI in healthcare refers to the application of complex algorithms and software to emulate human cognitive abilities in analysing, interpreting, and comprehending vast amounts of medical and healthcare data [4]. This empowers healthcare professionals to make more accurate diagnoses, develop personalized treatment plans, and ultimately improve patient outcomes. AI encompasses a wide range of technologies, including machine learning, natural language processing, robotics, and other advanced computational techniques that can assist in clinical decision-making, predictive analytics, and administrative tasks [4,5].

History of AI in healthcare

The concept of AI in medicine has evolved significantly since the 1960s, when pioneering expert systems like MYCIN and INTERNIST-1 were introduced to mimic human decision-making in clinical diagnostics [6,7]. Substantial progress occurred in the late 20th century, supported by the digitization of healthcare records and the availability of large-scale clinical datasets [6-8].

Notable milestones include the development of IBM’s Deep Blue in 1997, which demonstrated the feasibility of machine-based strategic reasoning. The early 2000s ushered in rapid growth in machine learning, particularly deep learning, fueling AI’s capacity to analyze medical images, genetic data, and complex patient information. The widespread adoption of electronic health records and improved data infrastructure further accelerated these advancements.

Today, AI systems are employed across diverse clinical domains, including medical imaging, robotic surgery, workflow optimization, and disease risk prediction. Recent breakthroughs such as federated learning, real-time wearable-based monitoring, and AI-driven mental health interventions have significantly broadened the scope of AI in healthcare. These developments are summarized in Table 1, highlighting their relevance and potential impact on clinical practice.

**Table 1:** Recent advances in AI in healthcare

Advance	Description	Significance	References
AI-Driven Personalized Medicine	AI now analyzes genetic and molecular profiles to customize treatments.	Improves precision in treatment for diseases like cancer, reducing side effects and improving outcomes.	[9]
AI-Powered Real-Time Monitoring	AI integrated with wearables for real-time health monitoring (e.g., glucose, heart rate).	Enhances patient safety through continuous monitoring and early detection of complications.	[10]
Federated Learning for Medical Data	Federated learning allows training AI models without sharing patient data.	Addresses privacy concerns by enabling collaboration without exposing sensitive data.	[11]
AI in Early Diagnosis of Rare Diseases	AI platforms now integrate multi-omics data to detect rare diseases early.	Provides faster, more accurate diagnoses in cases where traditional methods are inefficient.	[12]
AI-Based Mental Health Interventions	AI is being used to deliver mental health interventions via apps and chatbots.	Expands access to mental health care, offering personalized support based on behavioral data.	[13]

Why is there a need for guidelines

While AI holds immense potential to revolutionize healthcare delivery, its integration into clinical practice raises significant ethical concerns [14]. These concerns necessitate the development and implementation of clear ethical guidelines and frameworks. AI promises to improve efficiency, accuracy, and access to care, but it also introduces risks associated with machine-driven decision-making in high-stakes clinical environments. Ethical frameworks are essential to ensure that AI deployment respects patient rights, maintains clinical integrity, and upholds societal values.

Patient safety and well-being are primary concerns, as AI systems must prioritize minimizing potential risks and avoiding harm. Guidelines are needed to establish safety standards, risk assessment protocols, and oversight mechanisms to ensure clinical accuracy and prevent adverse outcomes.

Transparency and accountability are critical, particularly because AI algorithms, especially deep learning models, can be opaque and difficult to interpret. This lack of transparency raises concerns about accountability. Guidelines should promote clarity in AI development and decision-making processes, enabling stakeholders to understand how these systems function while holding developers and healthcare providers responsible for their actions. Data privacy and security must be safeguarded, as AI systems rely on vast amounts of sensitive patient data. Guidelines are necessary to ensure compliance with data protection regulations, secure storage, and ethical use of personal information to maintain patient trust.

Fairness and non-discrimination must be addressed, as AI algorithms can perpetuate or amplify existing biases if not carefully designed. Guidelines should promote fairness, inclusivity, and non-discrimination to ensure that AI systems do not disadvantage individuals or groups based on factors such as race, gender, or socioeconomic status.

Ethical principles and human values must guide AI integration, as healthcare raises complex questions about autonomy, dignity, beneficence, and justice. Guidelines should align AI development with established ethical principles and human rights, ensuring that technology respects societal norms. Human oversight remains indispensable, as even advanced AI should not replace clinical judgment. Ethical frameworks must require human involvement in decision-making to preserve patient autonomy and informed consent. Legal and regulatory compliance is another key consideration, as the rapid advancement of AI has outpaced existing laws. Guidelines can provide clarity on legal obligations, liability concerns, and regulatory expectations, ensuring responsible AI adoption in healthcare.

By establishing comprehensive ethical frameworks, stakeholders can address these concerns, foster public trust, and ensure that AI's benefits in healthcare are realized while minimizing risks and unintended consequences. A well-defined and enforceable ethical foundation not only mitigates potential harms but also supports the long-term sustainability of AI in medicine [14-16].

Guidelines available globally

Recognizing the ethical, legal, and social implications of AI in healthcare, various organizations and governments around the world have developed guidelines and frameworks to govern its responsible development and deployment. 

European Union guidelines on AI in healthcare

The European Union (EU) has emerged as a global leader in establishing ethical governance for artificial intelligence (AI), particularly in healthcare, where the stakes are exceptionally high. Recognizing both the transformative potential and inherent risks of AI, the EU has developed comprehensive guidelines to ensure that these technologies are deployed responsibly, transparently, and in alignment with fundamental human rights. The European Commission’s Ethics Guidelines for Trustworthy AI serve as a cornerstone, outlining key principles that prioritize human agency, technical robustness, privacy, fairness, and societal well-being [17-19]. These guidelines not only address immediate technical concerns but also consider broader ethical and legal implications, setting a benchmark for AI governance worldwide.

*Foundations of Trustworthy AI in Healthcare*

Developed by the High-Level Expert Group on AI, the EU’s framework mandates that AI systems must be lawful, ethical, and technically robust to earn public trust. Lawfulness ensures compliance with stringent regulations such as the General Data Protection Regulation (GDPR), safeguarding patient privacy and data security. Ethicality demands that AI aligns with core principles of fairness, accountability, and non-discrimination, preventing algorithmic biases that could exacerbate healthcare disparities. Technical robustness requires AI systems to perform reliably in real-world clinical settings, minimizing risks of errors that could harm patients [17-19].

A defining feature of the EU’s approach is its insistence on human agency and oversight. While AI can enhance diagnostic accuracy and treatment personalization, the guidelines emphasize that ultimate decision-making authority must remain with healthcare professionals. This safeguards patient autonomy and prevents over-reliance on automated systems. Additionally, transparency is a non-negotiable requirement; AI algorithms must be explainable, allowing clinicians and patients to understand how decisions are derived. This is particularly crucial in healthcare, where opaque "black-box" models could undermine trust and accountability.

*Preventing Discrimination and Ensuring Equity*

The EU guidelines explicitly address the risk of AI perpetuating systemic biases, mandating non-discrimination and fairness in algorithmic design. Given that AI models trained on historical data may inherit existing disparities, the framework calls for rigorous bias detection and mitigation strategies. This ensures that AI-driven healthcare solutions are inclusive, serving diverse populations equitably [17-19].

Beyond individual patient care, the guidelines also consider societal and environmental well-being, urging developers to assess long-term impacts, including sustainability and broader public health implications. Furthermore, accountability mechanisms are embedded into the framework, requiring clear lines of responsibility when AI systems are deployed, ensuring that errors or harms can be properly addressed.

*Extending Ethical Governance to Judicial and Broader Applications*

The EU’s commitment to ethical AI extends beyond healthcare, as seen in the European Ethical Charter on the Use of AI in Judicial Systems, developed by the European Commission for the Efficiency of Justice (CEPEJ). This charter reinforces similar principles, respect for fundamental rights, non-discrimination, quality assurance, transparency, and user control, ensuring that even in high-stakes legal contexts, AI remains subordinate to human judgment and ethical scrutiny [17].

By establishing these rigorous ethical standards, the EU not only mitigates risks but also fosters innovation within a trusted framework. The guidelines serve as a vital reference for policymakers, developers, and healthcare providers, ensuring that AI’s integration into medicine enhances, rather than undermines, patient safety, equity, and human dignity. As AI continues to evolve, the EU’s proactive governance model offers a blueprint for balancing technological advancement with ethical imperatives [17-19].

United States guidelines on AI in healthcare

The United States has developed a comprehensive yet flexible regulatory approach to govern artificial intelligence (AI) and machine learning (ML) applications in healthcare. Led by the Food and Drug Administration (FDA), this framework specifically addresses AI/ML-based Software as a Medical Device (SaMD) through policies that emphasize both innovation and patient safety [20,21].

*FDA Guidance on AI/ML-Based Software as a Medical Device (SaMD)*

The FDA's regulatory framework adopts a Total Product Lifecycle (TPLC) approach, recognizing that AI systems continuously evolve and require ongoing oversight [20-22]. This strategy begins with rigorous premarket review processes to validate safety and efficacy before deployment. Following approval, robust risk management protocols are implemented to identify and mitigate potential harms in clinical practice. The FDA mandates post-market surveillance to monitor real-world performance and requires transparency about algorithmic updates throughout the product lifecycle.

A key aspect of the FDA's approach is its recognition of AI's transformative potential in areas like early disease detection and precision diagnostics. The agency has established specialized policies for SaMD, categorizing AI/ML-based solutions according to risk level and requiring appropriate marketing applications. The framework also promotes Good Machine Learning Practices (GMLP), setting standards for data quality, model training, and clinical validation to ensure reliable performance [20-22].

*Ethical Considerations in Predictive Analytics Development and Implementation*

The U.S. guidelines emphasize ethical AI deployment, particularly for predictive analytics where algorithmic decisions can significantly impact patient care [20-23]. The framework addresses ethical concerns across four critical phases of development and implementation.

During data acquisition, the guidelines stress the importance of informed consent, which clearly explains data usage. They require representative datasets to prevent algorithmic bias and establish governance structures for proper data oversight. The model development phase incorporates fairness audits and maintains appropriate transparency about inputs and algorithms while balancing trade secrecy concerns.

When testing models in real-world settings, the guidelines mandate clear notice about predictive analytics usage and define liability frameworks for algorithmic outcomes. Regulatory oversight ensures compliance throughout implementation. Finally, during broad dissemination, the framework prioritizes equitable access to prevent healthcare disparities [24].

*Ethical Framework Integration*

The FDA integrates these ethical considerations throughout the AI/ML lifecycle, promoting fairness and transparency while ensuring patient safety [25]. By combining adaptive regulation with strong ethical safeguards, the U.S. framework provides a balanced approach to AI adoption in healthcare, fostering innovation while protecting patient rights and maintaining public trust [20-25].

Indian Council of Medical Research (ICMR) guidelines

The Indian Council of Medical Research (ICMR) has established comprehensive guidelines to govern the ethical implementation of AI in healthcare, recognizing both its transformative potential and associated risks [26-28]. These guidelines emphasize five foundational principles that collectively ensure responsible AI adoption while protecting patient rights and public health interests. Autonomy and human oversight form the cornerstone of ICMR's approach, mandating continuous human involvement in AI-assisted clinical decision-making. This principle safeguards physician judgment and preserves patient autonomy through maintained informed consent processes, ensuring AI serves as a decision-support tool rather than a replacement for human expertise [26-28].

Safety and risk minimization requirements call for the implementation of robust control mechanisms throughout the AI lifecycle. The guidelines mandate thorough risk assessments and validation protocols to identify and mitigate potential patient safety concerns before clinical deployment, with ongoing monitoring post-implementation. The trustworthiness and transparency principle demands that AI systems demonstrate reliability through rigorous testing while maintaining explainable decision-making processes. This dual requirement fosters trust among healthcare providers, patients, and regulators by ensuring AI outputs are both accurate and interpretable. Data privacy and confidentiality protections align with India's evolving digital privacy landscape, requiring strict data anonymization practices and compliance with emerging data protection legislation. The guidelines particularly emphasize safeguarding sensitive health information throughout all stages of AI development and deployment. Finally, the accountability framework establishes clear lines of responsibility among AI developers, healthcare institutions, and clinicians. This includes mechanisms for addressing adverse outcomes and ensuring recourse for patients affected by algorithmic decisions [26-28].

The ICMR guidelines pay special attention to equity concerns, mandating proactive measures to prevent algorithmic bias and ensure fair treatment of historically underrepresented populations. By addressing these critical issues, the framework aims to promote ethical AI integration that enhances healthcare delivery while protecting fundamental patient rights [26-28].

Australian guidelines on AI in healthcare

Australia's approach to AI governance in healthcare is anchored in its national Artificial Intelligence Ethics Framework, developed by the Australian Government to guide responsible AI development and deployment [29-33]. This comprehensive policy outlines eight core principles that collectively address the technical, ethical, and societal dimensions of healthcare AI implementation. The framework begins with Safety and Security requirements that mandate rigorous testing and protective measures throughout the AI lifecycle, from development through clinical deployment. These provisions work in tandem with Human Values protections that ensure AI systems respect fundamental rights and align with Australian societal norms [31-33].

Fairness provisions specifically address healthcare disparities by requiring inclusive design practices and bias mitigation strategies. This principle is supported by strong privacy protection standards that enforce stringent data governance protocols, which are particularly critical when handling sensitive health information. Transparency requirements call for clear documentation of AI system capabilities, limitations, and decision-making processes to build trust among stakeholders. These are reinforced by accountability mechanisms that define responsibility for AI outcomes across developers, healthcare providers, and institutions [31-33]. The framework emphasizes human oversight as a non-negotiable requirement for clinical AI applications, ensuring final decision-making authority remains with qualified healthcare professionals. Complementary security provisions focus on protecting AI systems from external threats that could compromise patient safety or data integrity [29-33].

In healthcare implementation, these principles translate to practical requirements for equitable service access, robust patient privacy safeguards, and clear accountability structures for AI-assisted medical decisions. Australia's approach aims to cultivate an ethical AI ecosystem that prioritizes individual welfare while promoting innovation through responsible, transparent, and inclusive technology deployment [31-33].

African guidelines on AI in healthcare

*Overview*

While Africa lacks a unified, continent-wide set of guidelines for the ethical use of AI in healthcare, various initiatives and collaborations are underway to address the ethical implications of AI deployment. These efforts aim to tackle critical challenges such as data privacy, personalized care, accessibility, and cultural considerations, ensuring that AI technologies benefit all segments of African society [34,35].

*Ethical Considerations and Initiatives*

Artificial intelligence holds significant promise for revolutionizing healthcare in Africa, providing solutions to pressing challenges such as reducing costs, improving access to care, and enhancing diagnostic accuracy. However, ethical integration of AI into healthcare systems is crucial to ensure that the benefits of AI outweigh its potential risks [34,35]. This section examines the ethical considerations surrounding the adoption of AI in Africa's healthcare sector, highlighting both opportunities and challenges.

Data privacy is a critical consideration, as AI systems rely on large datasets, including sensitive patient information. Ensuring robust data protection standards is necessary to prevent misuse and unauthorized access, thereby maintaining trust in AI technologies. Personalized care is another important area where AI can analyze individual health data to offer tailored treatment plans. Nonetheless, handling this data requires respecting patient confidentiality and obtaining informed consent. Ethical guidelines should ensure that AI is used to enhance personalized care without compromising patient rights. Accessibility to AI technologies must be ensured for all segments of society, particularly underserved and remote communities. Overcoming barriers such as infrastructure limitations, cost, and digital literacy is essential to prevent the exacerbation of existing healthcare disparities. Cultural considerations must be integrated into AI algorithms, accounting for Africa's linguistic and cultural diversity. AI systems should incorporate local languages and cultural nuances to ensure effectiveness and relevance across different healthcare settings.

*Opportunities and Challenges*

AI offers significant opportunities to enhance healthcare quality by enabling early disease detection and improving diagnostic accuracy. Healthcare professionals can leverage AI-enabled technologies to access vast datasets, leading to more informed clinical decisions and reduced misdiagnosis rates. This can substantially lower both health and financial burdens on patients. Furthermore, AI can automate administrative tasks, freeing up healthcare providers to focus more on direct patient care [34,35].

Despite these opportunities, several ethical challenges must be addressed for AI deployment in Africa's healthcare systems. Key concerns include safeguarding data privacy, ensuring the provision of personalized care, and guaranteeing equitable access to AI technologies. Developing and implementing ethical frameworks that are tailored to the African context is crucial to overcoming these challenges [34,35].

*Notable AI Initiatives in Africa*

Several AI initiatives are making significant strides in addressing healthcare challenges in Africa. DokiLink in Nigeria facilitates doctor-patient appointments and medical collaboration through AI-powered platforms, thereby enhancing the efficiency and accessibility of healthcare services [36]. The partnership between Novartis and Vodacom in South Africa leverages AI to improve access to healthcare services, especially in remote areas, showcasing the potential of AI to bridge healthcare gaps [37]. Apmis in Nigeria is a healthcare system that utilizes AI to optimize healthcare delivery and resource management, demonstrating tangible benefits in improving healthcare efficiency [38]. The Kenya Medical Supplies Agency's collaboration with IBM’s Watson uses AI to streamline healthcare logistics and improve resource management, highlighting the cost-saving potential of AI technologies [39].

These guidelines and frameworks share common themes, including the promotion of transparency, accountability, privacy protection, fairness, and human oversight. They also emphasize the importance of stakeholder engagement, collaboration, and continuous monitoring to ensure the responsible development and deployment of AI in healthcare. Table 2 summarizes the key ethical frameworks, principles, and unique features of AI guidelines developed by various countries and organizations, highlighting their application in healthcare and other sectors.

**Table 2:** Comparative overview of AI bioethics guidelines across countries and regions

Country/Region	Key Guideline Document	Overview	Key Ethical Principles	Unique Features
European Union [17-19]	Ethics Guidelines for Trustworthy AI	Developed by the High-Level Expert Group on AI, these guidelines emphasize principles for developing trustworthy AI, including human oversight, technical robustness, privacy, fairness, and societal well-being.	Human agency and oversight; Technical robustness and safety; Privacy and data governance; Transparency; Fairness and non-discrimination; Societal and environmental well-being; Accountability	Focus on human-centric AI and sustainability. Includes societal impact analysis. Regular updates in line with the EU AI Act.
United States [20-25]	FDA Guidance on AI/ML-Based SaMD	The FDA’s framework for regulating AI/ML in healthcare follows a Total Product Lifecycle (TPLC) approach. It balances innovation with safety by ensuring real-world monitoring, risk management, and premarket validation.	Risk-based approach; Transparency and real-world performance monitoring; Patient safety and data privacy; Informed consent; Accountability	Incorporates Good Machine Learning Practices (GMLP). Explicit focus on post-market surveillance and continuous learning systems.
India [26,28]	ICMR Ethical Guidelines for AI in Healthcare	These guidelines focus on integrating AI responsibly into healthcare with principles of autonomy, transparency, and accountability. They emphasize inclusiveness, safety, and privacy, while addressing the needs of vulnerable populations.	Human oversight; Risk minimization; Data privacy and confidentiality; Trustworthiness; Inclusivity and fairness; Accountability	Specific focus on marginalized populations and healthcare equity. Alignment with local laws like DISHA and PDP Bill.
Australia [29-33]	Australian AI Ethics Framework	Developed by the Australian Government, this framework provides ethical principles for AI deployment, focusing on safety, fairness, transparency, and privacy. It serves as a guide across various sectors, including healthcare.	Safety and security; Human values; Privacy protection; Transparency; Accountability; Human oversight; Fairness	Explicit focus on explainability and equitable access in rural and remote areas. Integration with National Digital Health Strategy.
United Kingdom [40]	UK AI Governance Framework	The UK emphasizes accountability, transparency, and ethical considerations in AI. Guidelines focus on balancing innovation with public trust, especially in healthcare applications like diagnostics and clinical decision support systems.	Public trust; Ethical use of data; Robustness and accuracy; Accountability; Transparency	Emphasis on public engagement and aligning AI with the National Health Service (NHS) principles.
Canada [41]	Pan-Canadian AI Strategy	Canada’s guidelines emphasize a human-centric and inclusive approach to AI. It focuses on ensuring that AI respects Canadian values, promoting fairness, transparency, and accountability.	Inclusivity; Transparency; Accountability; Data governance; Risk minimization	Comprehensive funding for ethical AI research and training. Focus on equitable healthcare delivery in diverse populations.
Japan [42]	Social Principles of Human-Centric AI	Japan’s guidelines promote the responsible and ethical use of AI by emphasizing harmony with society, respect for human dignity, and protection of personal information.	Human-centric AI; Respect for personal information; Safety; Accountability; Promotion of innovation	Strong alignment with cultural values. Explicit focus on coexistence with society and harmony with traditional healthcare practices.
China [43]	AI Governance Principles (Beijing AI Principles)	These guidelines emphasize the development of beneficial, fair, and secure AI systems. They also highlight the importance of fostering international cooperation and aligning AI use with societal values.	Harmony with societal values; Fairness and inclusivity; Transparency; Safety; International cooperation	Strong emphasis on state oversight and international collaboration. Alignment with national development priorities.
Global [44]	WHO Guidance on Ethics and Governance of AI in Health	The World Health Organization provides a global framework for ethical AI in healthcare. It highlights the need for equity, inclusiveness, and transparency, with a specific focus on addressing global health disparities.	Equity and inclusiveness; Human oversight; Transparency; Risk mitigation; Data privacy	Focus on global health equity and low-resource settings. Advocacy for responsible data-sharing practices in cross-border healthcare systems.

Guiding principles in AI development and validation

During the development phase of AI-based solutions, stakeholders, including academic researchers, industry sponsors, clinicians, and public health systems, play crucial roles. Ethical considerations such as informed consent, confidentiality, privacy, and non-discrimination are paramount. Data collection practices must be transparent, with measures like data encryption and anonymization employed to protect privacy [45,46]. Stakeholders are also responsible for ensuring accountability and liability. Compliance with regulations and standards for data security, privacy protection, and ethical conduct is essential. Continuous monitoring and improvement of data quality and algorithm performance enhance the accuracy and generalizability of AI technologies [45,46].

The development of ethical AI solutions for healthcare demands a multidisciplinary approach involving researchers, clinicians, policymakers, and technology developers. Academic institutions contribute methodological rigor through peer-reviewed research protocols, while industry partners provide essential technical infrastructure and scalability. Healthcare professionals ensure clinical relevance by validating that AI tools address real-world medical needs, and public health experts evaluate population-level impacts. This collaborative ecosystem must prioritize four foundational ethical requirements: meaningful informed consent processes that accommodate varying health literacy levels, comprehensive data protection measures, algorithmic fairness safeguards, and continuous performance monitoring.

Technical implementation requires robust security frameworks incorporating end-to-end encryption, differential privacy techniques, and strict access controls. The European Union's General Data Protection Regulation (GDPR) and similar frameworks globally have established important precedents for health data protection, though adaptations are needed for AI-specific challenges. Development teams should implement version control systems for algorithms and maintain detailed audit trails to support accountability. Regular bias audits using standardized fairness metrics can help identify and mitigate discriminatory patterns in AI outputs.

The validation phase focuses on establishing the safety, accuracy, and real-world performance of AI technologies. Robust validation ensures that AI technologies are fit-for-purpose and free from systemic biases. Multi-dimensional, multi-sectoral teams conduct objective assessments of AI performance, usability, and user experience. Frameworks like SPIRIT-AI and CONSORT-AI provide standardized approaches to validation, fostering trust among healthcare providers and recipients [47-49].

Addressing key challenges in healthcare AI implementation

Despite the immense potential of AI in healthcare, there are significant challenges that need to be addressed to ensure its responsible and ethical implementation. One major challenge is data quality and bias. AI algorithms are highly dependent on the quality and quantity of data used for training. Biases present in training data can lead to discriminatory or inaccurate outcomes [50,51]. To mitigate this, it is essential to implement robust data collection practices, ensure data diversity, and employ fairness metrics in AI models. Standardizing data formats and promoting interoperability across healthcare systems can further facilitate data sharing and collaboration [50,51].

Another critical challenge is transparency and explainability. The "black-box" nature of some AI algorithms, particularly deep learning models, makes it difficult to understand how they arrive at decisions, hindering trust and accountability [52]. Developing explainable AI (XAI) techniques that provide insights into decision-making processes and encouraging open-source development and collaboration are vital steps toward improving transparency [52].

Privacy and security also present significant concerns. AI systems in healthcare rely on vast amounts of sensitive patient data, and data breaches or unauthorized access can compromise patient privacy and security [53,54]. Addressing this requires robust cybersecurity measures, strict enforcement of data protection regulations, and education of healthcare professionals on data privacy best practices. Techniques such as anonymization and encryption should be developed to protect patient data while still enabling its use for AI development [53,54].

Human oversight and control remain fundamental. Although AI is a powerful tool, it should not replace human judgment and expertise. Healthcare professionals must maintain oversight and control over AI-driven decision-making processes [46,55,56]. Developing clear guidelines for human-AI collaboration, ensuring human oversight in critical decisions, and investing in training and education for healthcare professionals on AI capabilities and limitations are necessary steps [46,55,56].

Accessibility and equity are further important considerations. The benefits of AI in healthcare should be accessible to all, yet challenges such as limited infrastructure and digital literacy gaps risk creating disparities in access [57-59]. Implementing strategies that promote equitable access to AI-powered healthcare services, addressing factors like affordability, infrastructure, and digital literacy, and designing AI solutions that cater to diverse populations and the needs of low-resource settings are crucial [57-59].

Finally, the development of clear legal and regulatory frameworks is essential. The rapid evolution of AI technology necessitates governance frameworks that address issues such as liability, data ownership, and intellectual property rights [4,16,60]. Collaboration with policymakers and legal experts is needed to ensure comprehensive and adaptive regulations that can keep pace with technological advancements [60-62]. As AI continues to evolve, it brings forth new ethical challenges, including algorithmic bias, privacy concerns, and the transparency of AI systems. Recent advances have sought to mitigate these issues through novel approaches, as outlined in Table 3, which summarizes key challenges and their proposed solutions.

**Table 3:** Recent ethical challenges and solutions in AI in healthcare

Challenge	Description	Recent Solutions (2023-2024)
AI Algorithmic Bias [62]	AI models perpetuating existing healthcare biases	Development of bias detection algorithms and fairness metrics to mitigate inequitable outcomes.
Data Privacy in Decentralized AI [63]	Managing sensitive data in AI systems without central storage	Federated learning techniques allow AI training without exposing raw patient data to breaches.
Ethical Concerns in Autonomous AI [64]	AI making decisions with minimal human oversight	Introduction of "human-in-the-loop" and "human-on-the-loop" models to maintain human supervision.
Transparency of AI Decisions [65]	Difficulty in explaining complex AI decision-making processes	Advances in explainable AI (XAI) improve understanding of how AI reaches conclusions.

Future directions in healthcare AI

The next generation of healthcare AI is moving beyond pattern recognition to more sophisticated capabilities. Predictive analytics is evolving toward prescriptive systems that recommend personalized intervention strategies. These systems integrate multimodal data streams, including genomic, proteomic, and environmental factors, to generate comprehensive health insights. The integration of large language models into clinical workflows is creating new opportunities for automated documentation, patient communication, and knowledge synthesis.

Emerging applications demonstrate AI's potential to transform entire care pathways. In radiology, AI is progressing from detection assistance to comprehensive workflow optimization. For chronic disease management, continuous monitoring systems enable earlier intervention through subtle change detection. Drug discovery platforms are accelerating development timelines by orders of magnitude through in silico screening and trial optimization. These advances require parallel progress in validation methodologies and regulatory science.

The most significant breakthroughs will likely come from cross-disciplinary collaborations. Biomedical researchers working with materials scientists are developing novel biosensors for data collection. Clinicians collaborating with behavioral scientists are creating more effective human-AI interaction paradigms. Ethicists partnering with computer scientists are building fairness constraints directly into model architectures. These collaborations are producing AI systems that are not just technologically advanced but also clinically meaningful and socially responsible. Table 4 outlines the most anticipated future trends in AI that are expected to reshape the healthcare landscape.

**Table 4:** Future trends in AI in healthcare

Future Trend	Description	Potential Impact
AI in Preventive Healthcare [66]	AI will increasingly focus on prevention by predicting diseases before symptoms appear.	Reduces healthcare costs and improves outcomes by identifying risk factors and enabling early interventions.
Explainable AI (XAI) [65]	Development of more transparent and interpretable AI systems.	Enhances trust among healthcare professionals and patients, making AI decisions more understandable.
AI-Driven Drug Discovery [67]	AI algorithms will accelerate the drug discovery process by analyzing molecular data and simulating drug reactions.	Reduces the time and cost of developing new drugs, especially for complex diseases like cancer and Alzheimer’s.
AI for Global Health Equity [68]	AI solutions designed to address healthcare disparities in low-resource settings.	Expands access to advanced medical technologies in underdeveloped regions, improving global health equity.
AI and Genomics Integration [69]	AI will integrate with genomics for more personalized medicine, analyzing genetic data to predict disease risk and response to treatment.	Offers highly individualized care, improving treatment efficacy and reducing adverse effects.
AI in Remote and Virtual Care [70]	AI will enhance virtual health platforms, providing real-time diagnostics and personalized care remotely.	Increases access to healthcare services, particularly in rural or underserved areas, and improves patient monitoring.
AI in Mental Health Monitoring [71]	AI-driven platforms will continuously monitor mental health using behavioral, speech, and text analysis.	Provides early detection of mental health issues, enabling timely interventions and reducing suicide risks.

AI is being applied across a wide range of healthcare fields, with emerging applications that show great potential for revolutionizing diagnostics, treatments, and clinical decision-making. Table 5 highlights some of the most promising AI applications in specific healthcare domains.

**Table 5:** Future AI technologies impacting specific healthcare domains

Healthcare Domain	Future AI Technologies and Applications	Long-Term Impact
Surgery [72]	AI-enabled robotic surgery with real-time decision-making capabilities	Improves precision, reduces recovery times, and minimizes complications during complex surgeries.
Radiology [73]	AI algorithms for multi-modal image analysis (MRI, CT, ultrasound). AI-assisted radiology reports improving diagnostic speed and accuracy	Improves diagnostic accuracy by analyzing multiple imaging modalities simultaneously. Reduces human error and improves diagnostic efficiency, particularly in high-volume radiology departments.
Neurology [74]	AI for brain-computer interfaces (BCI) enabling direct communication with neural systems.	Revolutionizes treatments for neurological disorders like paralysis and Alzheimer’s, enhancing patient independence.
Pharmacology [75]	AI predicting drug interactions at the molecular level using quantum computing.	Leads to safer drug combinations and faster discovery of new therapeutic compounds.
Telemedicine [76]	AI-driven platforms providing fully virtual consultations with real-time diagnostics.	Reduces the need for physical consultations, offering broader access to healthcare services.
Chronic Disease Management [77]	AI-powered predictive models to manage chronic conditions like diabetes and heart disease.	Enables continuous and personalized monitoring, reducing hospital admissions and improving patient outcomes.
Oncology [78]	AI models predicting tumor response to immunotherapy	Enhances personalized cancer treatment planning, improving survival rates.
Cardiology [79]	AI systems predicting heart failure risk based on wearable device data	Real-time risk assessment enables timely interventions, reducing cardiac events and hospitalizations.
Mental Health [80]	AI chatbots providing cognitive behavioral therapy (CBT) interventions	Increases access to mental health care in underserved populations, offering timely psychological support.

The integration of AI into healthcare will require collaboration across multiple sectors, from public health to cybersecurity. Table 6 illustrates how interdisciplinary collaborations will shape AI-driven healthcare solutions, driving innovation and improving health outcomes.

**Table 6:** Future interdisciplinary collaborations in AI-driven healthcare

Sector	Future Role of AI	Potential Collaborative Impact
Healthcare [1]	AI will assist clinicians with diagnostics, personalized care, and treatment planning.	Improves patient outcomes, increases efficiency, and enhances decision-making in complex cases.
Public Health [81]	AI-driven models to predict and prevent outbreaks, optimize resource allocation.	Strengthens global public health responses to pandemics and health crises.
Bioinformatics [69]	AI will analyze genomic and proteomic data to create personalized treatments.	Leads to more effective, targeted therapies for genetic disorders and cancers.
Wearable Technology [10, 79]	AI algorithms will analyze data from wearable devices for continuous monitoring.	Empowers individuals to manage their health autonomously, preventing disease progression.
Cybersecurity [82]	AI-enhanced security systems to protect patient data and healthcare infrastructure.	Ensures data privacy, prevents cyberattacks on health systems, and increases trust in AI applications.

## Conclusions

The integration of AI into healthcare holds immense promise for transforming medical practice and improving patient outcomes. However, the establishment of robust ethical guidelines and governance frameworks is critical to ensure that AI technologies are developed and deployed responsibly. This paper has highlighted the pressing need to uphold ethical standards and prioritize patient safety as AI becomes increasingly embedded in medical applications. While AI offers significant benefits in enhancing diagnostic accuracy, treatment efficacy, and access to quality care, persistent concerns remain regarding privacy violations, the propagation of biases, and the erosion of human agency in clinical decision-making.

Addressing these ethical challenges is essential to fully realizing AI’s transformative potential in healthcare. Moving forward, active collaboration among stakeholders is crucial to promote ethical governance in the development and use of medical AI. Developers, healthcare providers, patients, and society at large must share responsibility for ensuring that ethical principles guide the evolution of AI-augmented healthcare systems. Ethical frameworks and management processes must be seamlessly integrated into established clinical workflows and reflect the shared values of all participants. Future research exploring the broad impacts of AI on global healthcare systems and individual health experiences will be indispensable. Effectively addressing these ethical challenges is essential for realizing the full potential of AI in healthcare. Collaboration among stakeholders, including developers, healthcare providers, patients, regulatory bodies, and society at large, is crucial to foster ethical governance in the design and application of medical AI.
